# Application and research progress of MultiBac: A review

**DOI:** 10.1097/MD.0000000000039333

**Published:** 2024-09-06

**Authors:** Zhangyang Feng, Jingjing Gao, Chunxin Jiang, Yunsen Li

**Affiliations:** a Institutes of Biology and Medical Science, Soochow University, Suzhou, China; b Department of Laboratory Medicine, The Affiliated Suzhou Hospital of Nanjing Medical University, Suzhou Municipal Hospital, Suzhou, China; c Institutes of Biology and Medical Science, Soochow University, Suzhou, China; d Institutes of Biology and Medical Science, Soochow University, Suzhou, China.

**Keywords:** BEVS, biotechnology, MultiBac, preclinical medicine, protein

## Abstract

**Background:**

Although the traditional *Escherichia coli* expression system has matured and is cost-effective, the posttranslation modifications of proteins expressed in eukaryotic cells differ significantly from those expressed in *E coli*. Insect cells have gradually entered the realm of researchers; however, the proteins synthesized by insect cells are somewhat different from those of mammals in terms of modification.

**Objective:**

Herein, we have introduced a relatively new method. MultiBac, We introduce the development process, characteristics, and applications of MultiBac technology. And provide new methods for basic researchers.

**Discussion:**

MultiBac has evolved into an indispensable tool in the fields of biotechnology and pharmaceuticals, facilitating the efficient production of recombinant proteins and the study of complex protein complexes. Furthermore, its development has benefited from the integration of synthetic biology techniques, providing additional versatility. But it also has some disadvantages.

**Conclusion:**

MultiBac technology is poised to become a key tool in unlocking the mysteries of the protein world, propelling the life sciences ever forward. But researchers should consider its limitations when selecting the most appropriate expression system for their specific needs.

## 1. Introduction

Research and development in academia and industry historically prioritized the study of small protein entities, often limited to fragments or domains.^[1–4]^ However, recent advancements in genomics, proteomics, and interactomics research have generated a wealth of biological data, revealing that cellular processes, especially in eukaryotes, are orchestrated by complex multiprotein assemblies rather than isolated proteins.^[5–8]^ These assemblies, which frequently comprise multiple subunits, pose challenges when producing individual eukaryotic proteins and protein complexes, including medically important ones like virus-like particles (VLPs), in *Escherichia coli* (*E coli*). The prokaryotic nature of *E coli* limits its efficiency in generating complex human protein specimens due to the significant distinctions between eukaryotic and prokaryotic cells.^[9]^ Eukaryotic cells and their constituent proteins exhibit higher complexity and require distinct folding, processing, and posttranslational modifications, such as glycosylation or phosphorylation,^[10–12]^ which cannot be adequately supported by the protein production machinery of *E coli*.^[13]^ Compared to the yeast expression system,^[14]^ the insect cell expression system exhibits lower background interference and greater posttranslational processing and modification capabilities. In comparison to mammalian cell expression systems, insect cells require less demanding cultivation conditions and equipment, making them easier to culture.^[15]^ Mammalian proteins expressed in insect cells can fold correctly and remain free of endogenous toxins. As a result, insect cell expression systems have come to the forefront as crucial tools in this context.^[16]^

Herein, we have introduced a relatively new method. MultiBac, a multigene expression system, is specifically designed for large-scale expression of multiple genes, making it highly suitable for protein production and research purposes.^[17]^ The inception of MultiBac can be traced back to a research initiative known as “MultiBac,” which was spearheaded by a team of scientists from the Max Planck Institute for Biophysical Chemistry in Germany in 2004.^[18]^ This project was dedicated to establishing an efficient multigene expression system. Over more than 4 decades, the baculovirus/insect cell expression system has evolved into a widely adopted production platform, bringing about significant transformations in research across academic and industrial laboratories. In the post-genome projects era, the focus has shifted towards recognizing multiprotein complexes as fundamental drivers of cellular functions. Readers can learn about the development process, features and applications of MultiBac technology through this review. It can provide a new option for basic research.

## 2. The development of MultiBac

### 2.1. MultiBac technology

MultiBac technology is a system specifically engineered to facilitate multigene expression in insect cells, primarily employed for protein production and research purposes. It is rooted in the baculovirus expression system, enabling the simultaneous and efficient expression of multiple genes within a single cell. Below, we provide an elaborate elucidation of the underlying principles of MultiBac technology^[17]^:

#### 2.1.1. Construction of MultiGene vectors

The core of MultiBac technology involves constructing multigene expression vectors, often referred to as multigene vectors. These vectors are designed to carry multiple target genes simultaneously. Each target gene has its own expression elements, such as promoters, terminators, selection markers, and more. These elements control the transcription and translation of genes.

#### 2.1.2. Vector assembly

The construction of multigene vectors typically utilizes molecular cloning techniques. Different target genes, along with their associated elements, are cloned into different positions within the multigene vector. This ensures that each gene is expressed at the right time and under the appropriate conditions. The construction of the vector is usually a systematic and precise process.

#### 2.1.3. Baculovirus vectors

MultiBac systems commonly employ baculoviruses as expression vectors. These baculoviruses have been engineered to accept genes from the multigene vector and express them in insect cells. Baculoviruses are highly effective at infecting insect cells, making them suitable for this system.

#### 2.1.4. Culturing insect cells15

Insect cells (such as Sf9 or Sf21 cells) are cultured to provide a suitable host environment. These cells are typically grown in culture dishes or bioreactors to ensure a sufficient quantity of cells for the experiments.

#### 2.1.5. Gene expression and protein production

Following baculovirus infection of the insect cells, the genes within the multigene vector undergo transcription and translation, facilitating the effective expression of multiple target proteins within a single cell. Subsequently, these proteins can then be harvested, purified, and subjected to further analysis or application.^[19]^

**Figure 1.**
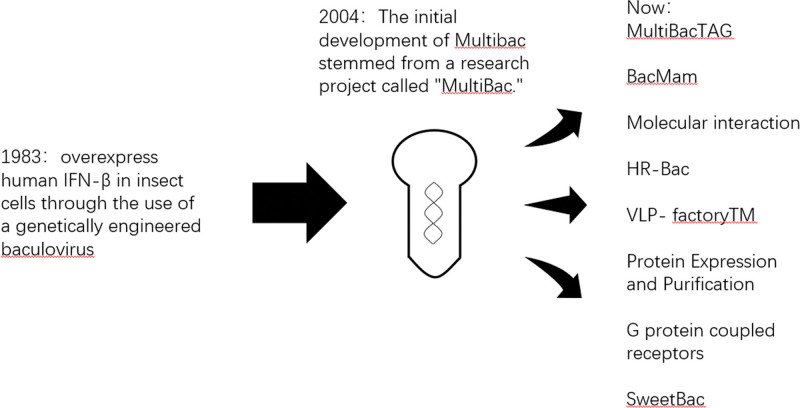
The origin and latest applications of MultiBac. (A) overview of the development of MultiBac technology: from the groundbreaking research on baculoviruses in 1983 to the proposal of MultiBac technology in 2004, and then to its current primary applications in 8 aspects.

In December 1983, a pivotal study was initially published, demonstrating a significant advancement in the overexpression of human IFN-β in insect cells through the use of a genetically modified baculovirus. This pioneering research revealed the potential of baculoviruses to generate substantial amounts of 2 proteins, polyhedrin and p10, by harnessing exceptionally powerful promoters. This pivotal discovery significantly contributed to the advancement of the baculovirus expression system.^[20]^ Over the past 4 decades, significant advancements have been made in improving the initial baculovirus expression vector system (BEVS). These improvements have enabled the customization of baculovirus vectors, the optimization of glycoprotein modifications in insect cell expression, and the scalability of cell culture processes. Consequently, this effective expression system has been widely employed to manufacture numerous recombinant proteins, including protein-based vaccines for both human and veterinary applications, available in today’s market. Recombinant baculovirus technology has also played a pivotal role in developing viral vectors derived from adeno-associated virus (AAV),^[21]^ resulting in the approval of the inaugural gene therapy treatment employing this approach. Tailored baculovirus expression vectors have shown their effectiveness in transferring and expressing foreign genes in mammalian cells, hinting at possible uses in gene therapy and cancer treatment. It is important to note that ongoing advancements in this versatile expression tool are driven by the integration of potent synthetic biology techniques, further expanding its capabilities. These innovative developments position the BEV system to maintain a crucial role in the life sciences, spanning both fundamental and applied research.^[20]^ Over more than 4 decades, the baculovirus/insect cell expression system has evolved into a widely adopted production platform, bringing about significant transformations in research across academic and industrial laboratories. In the post-genome projects era, the focus has shifted towards recognizing multiprotein complexes as fundamental drivers of cellular functions. Consequently, the MultiBac technology emerged as a response to this need. (Fig. 1).

### 2.2. The development of the MultiBac technology has gone through several significant stages

#### 2.2.1. Fundamental research (early 2000s)

The evolution of MultiBac technology began with in-depth research into the baculovirus expression system. Initially, the focus was on optimizing the baculovirus vector to enhance its expression efficiency in insect cells.

#### 2.2.2. Design of expression vectors (mid-2000s)

As understanding of the baculovirus expression system deepened, researchers started designing and constructing more flexible expression vectors. These vectors could accommodate multiple target genes, allowing scientists to simultaneously express multiple proteins.

#### 2.2.3. Study of protein complexes (early 2010s)

An important application of MultiBac technology is the efficient production of protein complexes. This technology enables scientists to investigate protein interactions and the structure of complexes, aiding in unraveling complex biological questions.

#### 2.2.4. Integration of synthetic biology techniques (mid-2010s)

With the rise of synthetic biology techniques, MultiBac technology also benefited from their integration. This made the technology more flexible and adaptable to evolving research needs.

#### 2.2.5. Expansion of applications (late 2010s to present)

The scope of applications for MultiBac technology has continued to expand. It is widely used not only in academic research but also in the fields of biotechnology and pharmaceuticals. It is employed for the efficient production of various recombinant proteins, including drugs and vaccines.

MultiBac primarily aimed to achieve simultaneous expression of multiple genes in insect cells using a baculovirus expression system. The research team successfully achieved efficient co-expression of multiple genes within a single cell by optimizing the construction of the baculovirus vector and designing and assembling expression vectors. The MultiBac has notably played a key role in providing access to previously elusive protein complexes, unveiling their intricate molecular structures and mechanisms in unprecedented detail. Moreover, BEVS has become an invaluable tool in the biotech and pharmaceutical sectors, enabling the production of complex biologics for disease prevention and therapy. The ongoing development of this versatile expression system continues to progress, especially with the introduction of powerful new synthetic biology techniques that are becoming increasingly accessible. With these impressive advancements, BEVS is poised to play an even more crucial and expanding role in the field of life sciences, encompassing both fundamental research and practical applications.^[22]^ MultiBac is currently employed in over a thousand laboratories worldwide, playing a crucial role in expediting ambitious research initiatives in both academia and industry.^[20,23–27]^

The baculovirus/insect cell system has proven highly effective for eukaryotic protein expression. In vaccinology and therapeutics, many products involve multiple protein subunits, which can be challenging for conventional baculoviral expression methods that lack efficient multigene expression strategies. However, the MultiBac technology, which merges a genetically modified *Autographa californica* nuclear polyhedrosis virus genome with purpose-built transfer vectors, offers a sophisticated approach to efficiently produce multi-subunit proteins in insect cells.^[28]^

## 3. Application of MultiBac

MultiBac was developed with specific goals in mind.^[29]^ First, it was designed to facilitate the expression of large eukaryotic multi-subunit complexes by accommodating multiple large genes and enabling their simultaneous expression. This capability is crucial for studying complex protein assemblies. Second, MultiBac aims to generate high-quality and homogeneous protein products that are well-suited for structural biology and a wide range of other applications. The system ensures the isolation of pure and well-defined protein complexes for detailed characterization. Third, MultiBac offers flexibility by allowing quick adjustments and revisions in expression experiments, enabling researchers to optimize their protocols efficiently. Finally, the system is user-friendly and robust, making it accessible to nonspecialists and promoting widespread adoption. Initially, MultiBac found primary usage in academic research settings. However, there has been an increasing demand for advanced production technologies like MultiBac in pharmaceutical research in recent years. This highlights the system’s effectiveness and potential for meeting the specific requirements of the pharmaceutical industry. By fulfilling these criteria, MultiBac serves as an invaluable tool for the expression and production of complex protein assemblies, catering to the needs of both academic and pharmaceutical research communities.^[17,30]^ Here we present some of the latest applications of MultiBac technology (Table 1).

**Table 1:** Example of the latest MultiBac application, including MultiBacTAG, BacMam, molecular interaction, HR-Bac, VLP-factory TM, protein expression and purification, G protein-coupled receptors, SweetBac.

Application	Example	References
MultiBacTAG	Producing such recombinant proteins using the tRNA/aminoacyl-tRNApyl synthetase pair or its mutants in insect cells	^[31]^
BacMam	The BacMam system is used for highly efficient gene editing in human cells through CRISPR/Cas9 delivery	^[32]^
Molecular interaction	A recombinant BV virus particle, designated as rBmBV-egfp-p64-3 × flag-gp64sp, was generated using the MultiBac baculovirus multigene expression system	^[33]^
HR-Bac	The HR-bac toolbox includes modified bacmids that express a fluorescent marker for tracking virus propagation and a collection of transfer vectors. The vectors feature either single or dual expression cassettes equipped with various affinity tags, enabling flexible utilization of expression units from MultiBac constructs. This design allows for versatile combinations of expression units from MultiBac constructs	^[34]^
VLP-factory TM	VLP-factory TM is a specialized MultiBac system designed for high-level expression of VLPs based on the influenza M1 capsid protein	^[35]^
Protein expression and purification	In vitro transcription assays were performed using RNA polymerase II enzyme (Pol II G) obtained from nuclear extracts, in combination with Mediator complexes of different compositions (head, middle, head + middle, head + middle + 14). All of these complexes were produced using the MultiBac system	^[36]^
G protein-coupled receptors	Established a functional receptor-G protein fusion complex, which can be readily purified at a large scale using ligand-based affinity chromatography	^[37]^
SweetBac	SweetBac is a system based on the MultiBac technology, designed to modify the glycosylation potential of insect cells. It achieves this by integrating the coding sequences of *Caenorhabditis elegans* N-acetylglucosaminyltransferase II and bovine β1,4-galactosyltransferase I together with the coding sequence of influenza hemagglutinin into the backbone of a baculovirus genome.	^[38]^

### 3.1. MultiBacTAG

The naturally occurring orthogonal pyrrolysine tRNA/aminoacyl-tRNApyl synthetase pair (tRNApyl/PylRS), commonly utilized in *E coli* and mammalian cell expression systems to produce recombinant proteins with novel chemical functionalities, has seen limited exploration in the BEVS. Koehler et al employed the traditional Bac-to-Bac system for the production of recombinant baculoviruses, but with an innovative inclusion of the tRNApyl/PylRS pair. They examined the production of recombinant proteins incorporating unnatural amino acids, exploring both in cis and in trans-configurations of the tRNApyl/PylRS pair concerning the target protein open reading frame (ORF). Their examination covered the design of transfer vectors and viral infection conditions. Genome sequence analysis conducted by Koehler et al conducted research on *Spodoptera frugiperda* and identified 8 U6 snRNA coding gene sequences that exhibited similarity to those found in other species. Notably, 6 of these U6 snRNA genes in insect cells possessed promoters that could effectively support genetic code expansion (GCE) in Sf21 insect cells. Therefore, in combination with these promoter elements, Koehler et al integrated the necessary components of *Methanosarcina mazei* tRNApyl/PylRS for GCE into a modified version of their MultiBac system. This technology can be used to the fluorescent labeling of target proteins and biologics using click chemistries, glycoengineering of antibodies, and structure–function studies of novel eukaryotic complexes using single-molecule Forster resonance energy transfer as well as site-specific crosslinking strategies.^[29,39]^ And, the researchers have completed sequencing 99.6% of the Sf21 genome. It will allow for further genetic engineering of this cell line for protein production using GCE.^[40]^

### 3.2. BacMam

Leveraging the progress in CRISPR/Cas9 technology and the capacity of BEVS to transport sizable cargo, the BacMam system has found utility in achieving highly effective gene editing in human cells through CRISPR/Cas9 delivery.^[31]^ BacMam plays a significant role as a CRISPR/Cas9 delivery system in human cells, enabling a variety of applications, including CRISPR-mediated protein tagging (e.g., using CRISPR/Cas9, HiBiT can be rapidly and efficiently integrated into the genome to serve as a reporter tag for endogenous proteins),^[32]^ simultaneous delivery of CRISPR/Cas9 gene editing machinery and drug release as nanodevices for biomedical therapy (e.g., mesoporous silica nanoparticles (MSNs), able to deliver the CRISPR/Cas9 machinery to edit gasderminD (GSDMD), a key protein involved in inflammatory cell death, and the anti-inflammatory drug VX-765 (GSDMD45CRISPR-VX-MSNs), were prepared. Nanoparticles allow high cargo loading and CRISPR-Cas9 plasmid protection and, thus, achieve the controlled codelivery of CRISPR-Cas9 and the drug in cells. Nanoparticles exhibit GSDMD gene editing by downregulating inflammatory cell death and achieving a combined effect on decreasing the inflammatory response by the codelivery of VX-765),^[33]^ accurate positioning of extensive multifunctional DNA circuits (e.g., synthetic virus-derived nanosystems (SVNs) capable of inserting complex DNAs into genomes, at base pair precision),^[34]^ accurate and specific gene delivery to mammalian cells.^[35]^

### 3.3. Molecular interaction

A recombinant BV virus particle, designated as rBmBV-egfp-p64-3 × flag-gp64sp, was produced utilizing the MultiBac baculovirus multigene expression system within the framework of *Bombyx mori* nucleopolyhedrovirus (BmNPV), a well-known model baculovirus with adverse effects on sericulture. This innovative approach not only allows for the identification of putative proteins implicated in BmNPV host invasion and propagation but also establishes a foundation for pinpointing potential receptor proteins involved in BmNPV interactions.^[36]^

### 3.4. HR-Bac

In order to support parallel expression screening and improve the potential for high-throughput applications, scientists have introduced an open-source toolbox for multigene expression that utilizes homologous recombination. This toolbox simplifies the process of creating recombinant baculoviruses into a 1-step procedure, greatly reducing the time needed for cloning and protein production to only 2 weeks. Referred to as the HR-bac toolbox, it includes modified bacmids created for expressing a fluorescent marker to monitor virus propagation, accompanied by a flexible collection of transfer vectors. These vectors incorporate single or dual expression cassettes, each equipped with various affinity tags, offering versatile combinations derived from MultiBac constructs. Importantly, the cost of virus generation using the HR-bac toolbox is notably economical overall.^[37]^

### 3.5. VLP-factory TM

VLP-factory TM is a specialized MultiBac system tailored for the high-level expression of VLPs utilizing the influenza M1 capsid protein. The viral backbone includes a gene encoding M1 from the H1N1 influenza strain, along with a gene encoding the mCherry fluorescent protein. This allows for monitoring virus amplification and VLP production during cell culture. This system has demonstrated its efficiency in generating various influenza VLPs, including those with mutations in the potentially immune-suppressive domain (ISD) located within the influenza surface protein hemagglutinin (HA). ISDs were originally identified in retroviruses, and it is proposed that influenza HA also possesses an ISD that partially overlaps with its fusion peptide region. This ISD could potentially influence innate immune responses in macrophages and dendritic cells. Considering its position within the structurally sensitive region of HA essential for membrane fusion, any alterations aimed at boosting antigenicity while eliminating immune suppression should be meticulously designed to preserve HA’s membrane fusion functionality. To pinpoint such mutants, it was necessary to create an array of influenza VLPs with randomized amino acids at particular positions within the ISD. These derived influenza VLP variants, consisting of wild-type HA or HA ISD mutants, can undergo functional assessments for in vitro membrane fusion activity and in vivo immune-suppressive activity in animal models. Influenza VLPs with ISD mutations represent a potential as hyper-immunogenic antigens, offering the prospect of robust and broadly protective influenza vaccines. Mutations in the HA ISD, found to be highly effective in eliminating immune suppression and enhancing HA antigenicity, are expected to elicit more potent neutralizing antibody responses when compared to unaltered HA. This process details the preparation of M1-based influenza VLPs incorporating HA with various ISD mutations. Similarly, the VLP-factory system can effectively present various viral or nonviral glycoproteins, their domains, structured epitopes, or desired target proteins on the surface of enveloped VLPs. This is accomplished by utilizing a native or heterologous transmembrane domain for secure anchoring.^[38]^

The *Bombyx mori* cytoplasmic polyhedrosis virus (BmCPV) is classified in the Reoviridae family and falls under the Cypovirus genus. It is characterized by a single-layer capsid and contains double-stranded RNA (dsRNA). Prior studies have shown that the major capsid shell protein (CSP) of BmCPV possesses the remarkable ability to self-assemble into VLPs. Cryo-electron microscopy investigations of BmCPV virions have revealed a tightly interacting region between the CSP and another capsid protein called the large protrusion protein (LPP). This interaction is crucial for stabilizing the capsid structure. Ren et al utilized the Ac-MultiBac multigene baculovirus expression system to produce VLPs. These VLPs were composed of CSP alone and in combination with LPP, demonstrating their co-assembly capability. The experimental outcomes indicate a robust interaction between CSP and LPP, reminiscent of a “Plug and Display” model where CSP acts as the “catcher” and LPP as the “tag.” This interaction leads to the formation of VLPs sharing structural similarities with native BmCPV virions. Furthermore, it demonstrates the feasibility of creating VLPs using both BmCPV capsid proteins, laying the groundwork for the development of BmCPV-based VLPs as a novel biological material for displaying foreign proteins.^[41]^

### 3.6. Protein expression and purification

In vitro transcription assays were conducted using RNA polymerase II enzyme, specifically Pol II (G), which was purified from nuclear extracts. Additionally, mediator complexes with various compositions, including head, middle, head + middle, and head + middle + 14, were generated utilizing the MultiBac system. The results of these assays mark a significant milestone, showcasing for the first time that both the recombinant Mediator complex with 15 subunits and the complete native Mediator complex consisting of 30 subunits displayed complete transcription recovery in the presence of Pol II (G). Furthermore, the findings indicate that the core Mediator’s functionality lies in its capacity to interact with Pol II (G), ultimately leading to the recruitment of Pol II to promoters.^[42]^

### 3.7. G protein-coupled receptors

G protein-coupled receptors (GPCRs) play a pivotal role in recognizing a wide range of stimuli to initiate diverse cellular responses. Their capacity to fulfill this diverse array of functions is made possible through modular interactions with intracellular transducers like heterotrimeric G proteins and arrestins. However, constructing a particular GPCR–G protein complex for structural and functional investigations remains a formidable task due to the limited affinity of their interaction. In a recent advancement, scientists improved fusion constructs of the Gα subunit of heterotrimeric G proteins and engineered variants of the rat neurotensin receptor 1 (NTR1). These constructs were coexpressed and assembled in vivo with Gβ and Gγ utilizing the baculovirus-based MultiBac system. This innovative approach led to the formation of a functional receptor–G protein fusion complex, which can be effectively purified on a large scale using ligand-based affinity chromatography. This development opens up new possibilities for investigating the structural and functional aspects of GPCR–G protein interactions.^[43]^

### 3.8. SweetBac

The capability to seamlessly incorporate ORFs encoding different proteins (or even RNA molecules) within a single baculovirus particle serves as an excellent platform for generating various baculoviruses that can confer unique glycosylation capabilities to the same expression host.^[44]^ This innovative platform is referred to as the SweetBac system.^[11]^ An extension of the MultiBac technology, the SweetBac system focuses on altering the glycosylation capabilities of insect cells used as expression hosts, allowing for versatile glycosylation of target proteins within these cells. Dieter Palmberger introduced sequences encoding *Caenorhabditis elegans* N-acetylglucosaminyltransferase II and bovine b1,4-galactosyltransferase I into the baculovirus genome to establish SweetBac. The adapted SweetBac virus was subsequently used to synthesize the human HIV anti-gp41 antibody 3D6 by incorporating ORFs for both heavy and light chains into the SweetBac genome. The simultaneous expression of target genes and glycosyltransferases resulted in a decrease in the antibody’s secreted yield. Nonetheless, the overall expression levels, especially in the newly established Tnao38 cell line, were similar to those attained with transient expression in mammalian cells. To evaluate SweetBac’ s ability to generate the 3D6 antibody with N-glycan structures resembling those in mammalian cells, Dieter Palmberger conducted SDS-PAGE and assessed the presence of terminal galactose using *Ricinus communis* agglutinin I. The mammalianized versions of 3D6 demonstrated specific binding to the lectin, confirming their functional integrity. The SweetBac system, an extension of MultiBac, demonstrates its potential for efficient production of mammalianized proteins in insect cells, offering advantages in terms of glycosylation patterns and antibody functionality.^[45–48]^

As an alternative to cell engineering, some researchers suggest modulating gene expression by introducing arrays of relevant proteins into the baculovirus using the MultiBac system as a platform. This approach provides increased flexibility since cells do not bear an additional metabolic burden when uninfected, thus mitigating the risk of cellular instabilities. Furthermore, the expression levels of co-factors are directly proportional to the number of infectious particles per cell. As a result, higher multiplicities of infection lead to a higher dosage of chaperones or glycosyltransferases, for example. It is worth highlighting that this system is not restricted to the expression of beneficial proteins; it can also be utilized for knock-downs by incorporating antisense RNAs to decrease undesired enzymatic activities. However, it is important to note that complete genome sequences or comprehensive sets of cDNAs required for identifying relevant target genes are not currently accessible for these insect cell lines. Nevertheless, ongoing projects are actively addressing these limitations.^[28]^

In addition to the examples mentioned above, in recent years, MultiBac technology has found numerous hotspots of application in the field of life sciences. Here are some of the prominent application areas. Protein complex research: MultiBac technology is widely employed for the efficient production of protein complexes composed of multiple proteins. This is crucial for deciphering cell signaling, protein–protein interactions, and complex structures. Vaccine and drug development: MultiBac technology can be used to efficiently produce candidate molecules for vaccines and drugs. It provides pharmaceutical companies with an effective method to generate recombinant proteins used in vaccine production and drug screening. Synthetic biology research: with the rise of synthetic biology, MultiBac technology has also benefited from advancements in this field. It is used to engineer proteins and metabolic pathways with novel functions, contributing to the design of new biomaterials and biotechnological applications. Cancer research: researchers utilize MultiBac technology to express and study proteins related to cancer, allowing for a deeper understanding of the biological characteristics of cancer cells and potential therapeutic targets. Structural biology: MultiBac technology is applied to produce a large quantity of protein samples for structural biology studies such as X-ray crystallography and electron microscopy. This aids in elucidating the 3-dimensional structures of proteins. Metabolic engineering: In fields like biofuel and chemical production, MultiBac technology is used to modify microbial metabolic pathways, enhancing production efficiency, and product yields. In summary, MultiBac technology has versatile applications across various domains, and the choice of hotspot areas depends on specific research and application needs. Its efficiency and versatility make it an essential tool in life science research and applications.

## 4. Discussion

The development of MultiBac technology encompasses the optimization of baculovirus vectors, the design and assembly of expression vectors, and the efficient production of protein complexes. This technology not only offers a means to express multiple proteins but also aids in deciphering the mechanisms of intricate protein interactions and structures. Over time, MultiBac has evolved into an indispensable tool in the fields of biotechnology and pharmaceuticals, facilitating the efficient production of recombinant proteins and the study of complex protein complexes. Furthermore, its development has benefited from the integration of synthetic biology techniques, providing additional versatility. In summary, the developmental journey of MultiBac technology represents a significant advancement in the field of baculovirus expression systems, offering scientists more tools to explore complex questions in the life sciences. In the realms of scientific research and biotechnology, MultiBac technology undoubtedly represents an exciting breakthrough. Its unique capabilities and properties open up wide-ranging prospects for the future of scientific research and applications.

### 4.1. MultiBac’s future diverse development directions

Revolutionizing structural biology. MultiBac technology is poised to revolutionize the field of structural biology. It allows scientists to delve deeper into the structures of complex proteins, leading to a profound understanding of cellular and life processes. This is likely to drive innovation in drug design and therapies, as a deeper comprehension of protein structures forms the basis for targeted drug development. Accelerating drug discovery: MultiBac technology offers new possibilities in drug development. It can produce proteins that were once considered difficult to prepare, opening up new avenues for disease research. This has the potential to accelerate the pace of drug discovery, offering breakthroughs in the treatment of rare diseases and personalized medicine. The future of protein engineering: MultiBac technology holds immense potential in the field of protein engineering. Scientists can design and produce customized proteins for industrial applications, biofuels, and biopharmaceuticals. This is likely to drive innovation and growth in many industries. A revolution in gene therapy: gene therapy is a key area of future medicine. MultiBac technology efficiently prepares the proteins required for gene therapy, offering new hope for the treatment of genetic diseases and cancers. Catalyst for cellular engineering: applications of MultiBac technology contribute to advancements in cellular engineering. It can be used to produce specific proteins, thereby making strides in synthetic biology and regenerative medicine. This could pave the way for new cellular therapies.

### 4.2. Applications and significance

Accelerating scientific research: MultiBac technology will expedite scientific research. Scientists will be able to access high-quality proteins more easily, facilitating in-depth studies of cell and protein functions, unlocking many mysteries in the life sciences. Medical advancements: in the medical field, MultiBac technology offers opportunities for disease treatment and drug development. It can aid in producing the proteins necessary for treatment, thereby advancing personalized healthcare and customized therapies. Biotechnological innovations: MultiBac technology will bring about revolutionary innovations in various biotechnology sectors, including industrial production, energy generation, and environmental protection. It is poised to become a key engine for biotechnological advancements. Education and training: the application of MultiBac technology will become a significant component of biological science education and training. Young scientists and researchers will benefit from learning and applying this advanced technology.

### 4.3. MultiBac technology

While powerful and versatile, does come with certain limitations and drawbacks. Here are some of the disadvantages of MultiBac technology: dependency on viral vectors: MultiBac relies on baculoviruses as expression vectors. Reliance on viruses might not be suitable for all applications, especially in environments where virus usage is restricted or undesirable. Complexity of glycosylation control: while MultiBac is used for glycosylation studies, it may not provide the level of control over complex glycosylation patterns that some applications require. Achieving precise glycosylation patterns can be challenging. Posttranslational Modifications: MultiBac primarily focuses on nucleotide-level expression of proteins. Certain posttranslational modifications may be limited in insect cells. Cell toxicity and stability: baculovirus infection can potentially lead to cell toxicity, affecting cell stability and growth. Additionally, maintaining viral stocks may require regular maintenance and expansion. Scale-up challenges: while MultiBac is well-suited for laboratory-scale protein production, scaling up to industrial levels may require additional optimization and resources. Cost: the construction and maintenance of MultiBac systems can be cost-intensive compared to some other expression systems.

## 5. Conclusion

In conclusion, the future of MultiBac technology looks promising. It will foster scientific progress, improve healthcare, drive biotechnological development, and create new opportunities across various domains. However, researchers will need to continually optimize and refine this technology to overcome potential challenges and ensure it fully realizes its enormous potential. MultiBac technology is poised to become a key tool in unlocking the mysteries of the protein world, propelling the life sciences ever forward. But researchers should consider these limitations when selecting the most appropriate expression system for their specific needs.

## Author contributions

**Supervision:** Yunsen Li

**Validation:** Chunxin Jiang

**Writing – original draft:** Zhangyang Feng

**Writing – review & editing:** Jingjing Gao, Yunsen Li
